# Influence of noninvasive brain stimulation on connectivity and local activation: a combined tDCS and fMRI study

**DOI:** 10.1007/s00406-023-01666-y

**Published:** 2023-08-19

**Authors:** Luise Victoria Claaß, Annika Hedrich, Janis Reinelt, Bernhard Sehm, Arno Villringer, Florian Schlagenhauf, Jakob Kaminski

**Affiliations:** 1 Department of Neurology, Max-Planck Institute for Human Cognitive and Brain Sciences, Stephanstraße 1, 04103 Leipzig, Germany; 2 Department of Psychiatry and Neurosciences CCM, Charité-Universitätsmedizin Berlin, Corporate member of Freie Universität Berlin, Humboldt-Universität zu Berlin and Berlin Institute of Health, Charitéplatz 1, 10117 Berlin, Germany; 3 Department of Neurology, Martin Luther University of Halle-Wittenberg, Ernst-Grube-Str. 40, 06120 Halle, Germany; 4 Day Clinic for Cognitive Neurology, University Hospital at the University of Leipzig, Liebigstraße 16, 04103 Leipzig, Germany; 5 Berlin School of Mind and Brain, MindBrainBody Institute, Humboldt-Universität zu Berlin, Unter den Linden 6, 10999 Berlin, Germany; 6 Bernstein Center for Computational Neuroscience, Humboldt-Universität zu Berlin, Unter den Linden 6, 10099 Berlin, Germany; 7 Department of Psychiatry and Psychotherapy, Charité–Universitätsmedizin Berlin, Campus Mitte, Charitéplatz 1, 10117 Berlin, Germany

**Keywords:** tDCS, fMRI, Working memory, Executive function

## Abstract

The effect of transcranial direct current stimulation (tDCS) on neurobiological mechanisms underlying executive function in the human brain remains elusive. This study aims at examining the effect of anodal and cathodal tDCS over the left dorsolateral prefrontal cortex (DLPFC) in comparison with sham stimulation on resting-state connectivity as well as functional activation and working memory performance. We hypothesized perturbed fronto-parietal resting-state connectivity during stimulation and altered working memory performance combined with modified functional working memory-related activation. We applied tDCS with 1 mA for 21 min over the DLPFC inside an fMRI scanner. During stimulation, resting-state fMRI was acquired and task-dependent fMRI during working memory task performance was acquired directly after stimulation. *N* = 36 healthy subjects were studied in a within-subject design with three different experimental conditions (anodal, cathodal and sham) in a double-blind design. Seed-based functional connectivity analyses and dynamic causal modeling were conducted for the resting-state fMRI data. We found a significant stimulation by region interaction in the seed-based ROI-to-ROI resting-state connectivity, but no effect on effective connectivity. We also did not find an effect of stimulation on task-dependent signal alterations in working memory activation in our regions of interest and no effect on working memory performance parameters. We found effects on measures of seed-based resting-state connectivity, while measures of effective connectivity and task-based connectivity did not show any stimulation effect. We could not replicate previous findings of tDCS stimulation effects on behavioral outcomes. We critically discuss possible methodological limitations and implications for future studies.

**Supplementary Information:**

The online version contains supplementary material available at 10.1007/s00406-023-01666-y.

## Introduction

Over the past years, there has been a growing neuroscientific and clinical interest in noninvasive brain stimulation techniques, with transcranial direct current stimulation (tDCS) as an important example [1, 2]. So far it has been shown that cortical stimulation of the DLPFC [3] and that of the primary sensorimotor cortices [4] alter resting-state connectivity and can enhance working memory processing [5]. There is evidence that anodal tDCS results in an increase in activity and excitability, whereas cathodal tDCS leads to a reduction of excitability [6], whereby recent studies show nonlinear and heterogeneous effects of cathodal tDCS in cognitive tasks [7–9].

Baudewig and colleagues were the first to combine the application of tDCS and neuroimaging techniques, using the blood oxygenation-level-dependent (BOLD) signal from functional magnetic resonance imaging (fMRI) [10]. Simultaneous tDCS stimulation and fMRI measurement is a highly promising method to better understand the effects of stimulation techniques [11]. Working memory performance has been shown to activate a robust network in dorsolateral prefrontal cortex [12, 13]. The working memory performance can be altered through anodal tDCS [5]; however, the underlying neurobiology of the behavioral stimulation effects remains elusive. Moreover, altered network connectivity has been suggested to be important in various neuropsychiatric disorders, e.g., in schizophrenia where deficits in fronto-parietal effective connectivity might underlie cognitive deficits [14, 15] and has been proven to be a promising marker for clinically relevant classification and clustering [16]. Finding similar patterns in neurobiological effects of tDCS as compared to clinical findings would be an important link informing mechanistic insight for putative treatment effects. To our best knowledge, in vivo investigation of effects of tDCS on neurobiological mechanisms underlying executive function in the human brain remains elusive.

In this study, we planned to investigate the influence of tDCS on neurobiological correlates in the prefrontal–parietal network during resting-state fMRI and lasting effects on working memory activation as well as effects on working memory performance. Therefore, we applied tDCS over left dorsolateral prefrontal cortex (DLPFC), while we recorded fMRI signals during rest and subsequent performance of a working memory task. The fMRI signal during rest provided insight into resting-state connectivity alterations by the ongoing stimulation, while the fMRI signal during task performance allowed us to test for changes in task-related local activity directly after stimulation. In a within-subject design, we measured these parameters during three randomized and counterbalanced conditions: (1) an active anodal stimulation, (2) a cathodal stimulation and (3.) a control condition with sham stimulation.

We hypothesized perturbed fronto-parietal resting-state connectivity measures during stimulation and altered functional activation after the different stimulation sessions in line with increased working memory performance during anodal and decreased performance during cathodal stimulation in comparison with sham stimulation.

## Methods

Thirty-six healthy young volunteers (mean age = 26.97 years, sd: 3.53, 18 women) were recruited in this double-blind, sham-controlled and randomized study. A statistical power analysis was performed for sample size estimation, based on data from a meta-analysis by Hill et al. (including 16 studies), comparing effects of anodal tDCS on working memory in healthy and neuropsychiatric populations [5]. We estimated the necessary sample size using GPower 3.1 [17] for a repeated-measures ANOVA with an alpha = 0.05 and power = 0.80. The projected sample size needed with the effect size of 0.21 in behavioral effects of tDCS derived from Hill et al. was estimated at *N* = 35 for a within group comparison, and thus, the target sample size of 36 was considered a sufficient sample size to reach 80% power. All subjects were right-handed, as assessed with the Edinburgh Handedness Inventory [18]. Prior to study inclusion, written informed consent was obtained from all subjects according to the Declaration of Helsinki. The study was approved by the ethics committee of the University of Leipzig.

All subjects underwent a comprehensive neurological examination to screen for potential exclusion criteria. Exclusion criteria were previously diagnosed psychiatric and neurological disorders, severe acute or chronic conditions and malignancies, in particular diseases of the thyroid gland, intake of medication except for contraceptives, alcohol or drug abuse, previous neurosurgical procedures, seizures and proneness to seizures and pregnancy. Only subjects who met the protocol criteria and had no contraindications to tDCS or MRI measurements were included for participation.

### Experimental design

For this study, a within-subject design was deployed. Healthy controls were measured repeatedly under three different experimental conditions, namely anodal, cathodal and sham stimulation. To avoid sequence effects, the stimulation types anodal, cathodal and sham stimulation were counterbalanced. All subjects underwent three tDCS sessions on three separate days with at least a one-week interval between stimulations to avoid carryover effects.

**Fig. 1:**
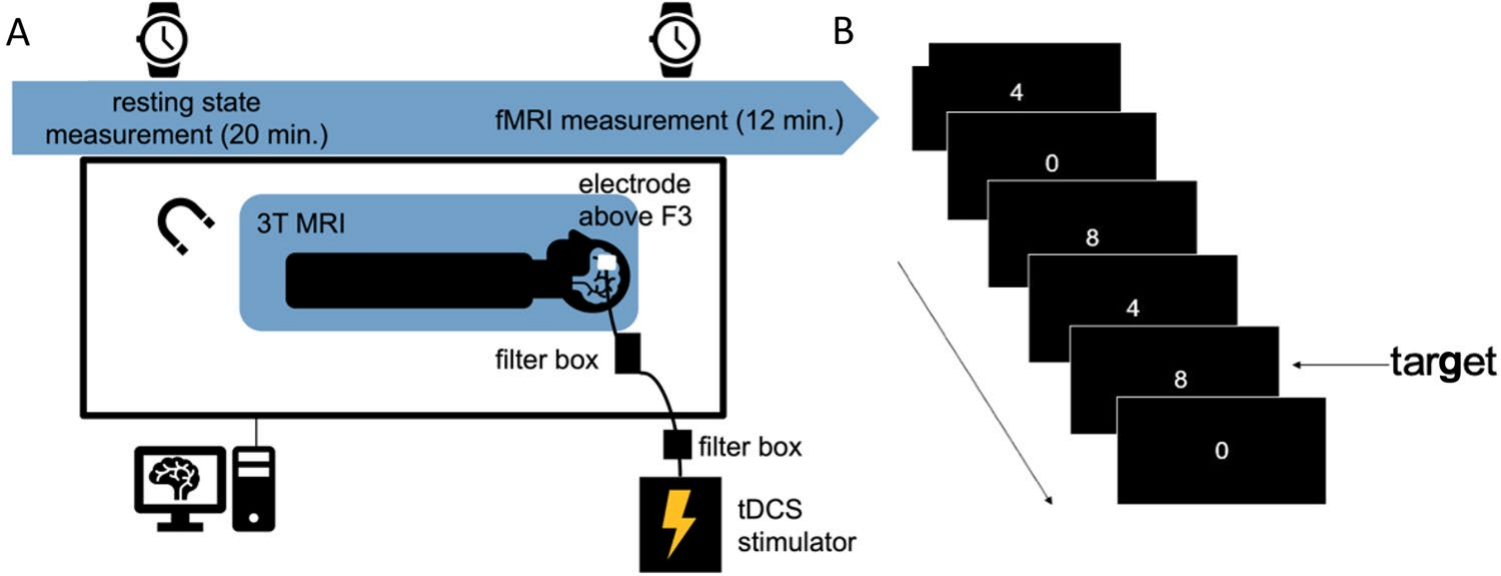
**A** Setup of stimulation and concomitant scanning procedure. The tDCS stimulator was located outside of the MR cabin and set at 1 mA. The cables connecting electrodes and stimulator passed on their way through the wall a radio frequency filter to reduce possible artifacts. Two filter boxes were positioned between electrodes and stimulator, one of them inside the MR cabin and the other one immediately outside. For scanning, a standard 32-channel head coil was used inside a 3 T scanner. First, a resting-state fMRI scan during stimulation was conducted after which a subsequent fMRI scan during the performance of a working memory task took place. **B** Setup of the 2-back experiment. During this task, subjects are seeing digits from zero to 9 for 500 ms with an inter-stimulus interval of 300 ms while lying supine inside the scanner. The participants are instructed to press a button each time a number is displayed which is equivalent to second to the last

A resting-state fMRI scan was conducted during each tDCS procedure with a subsequent fMRI scan during the performance of a working memory task. In order to blind the participants, the sham stimulation as baseline parameter included 30 s of ramping in, 30 s of stimulation and 30 s of ramping out. This produces a transient tickling sensation according to the sensation in the beginning of active stimulation, producing sufficient blinding [19]. The stimulation duration was 21 min including 30 s of ramping before and 30 s of ramping after stimulation. A person experienced with handling tDCS stimulators, who was not the experimenter, set up the stimulator (Fig. 1A).

### Transcranial direct current stimulation

**Fig. 2:**
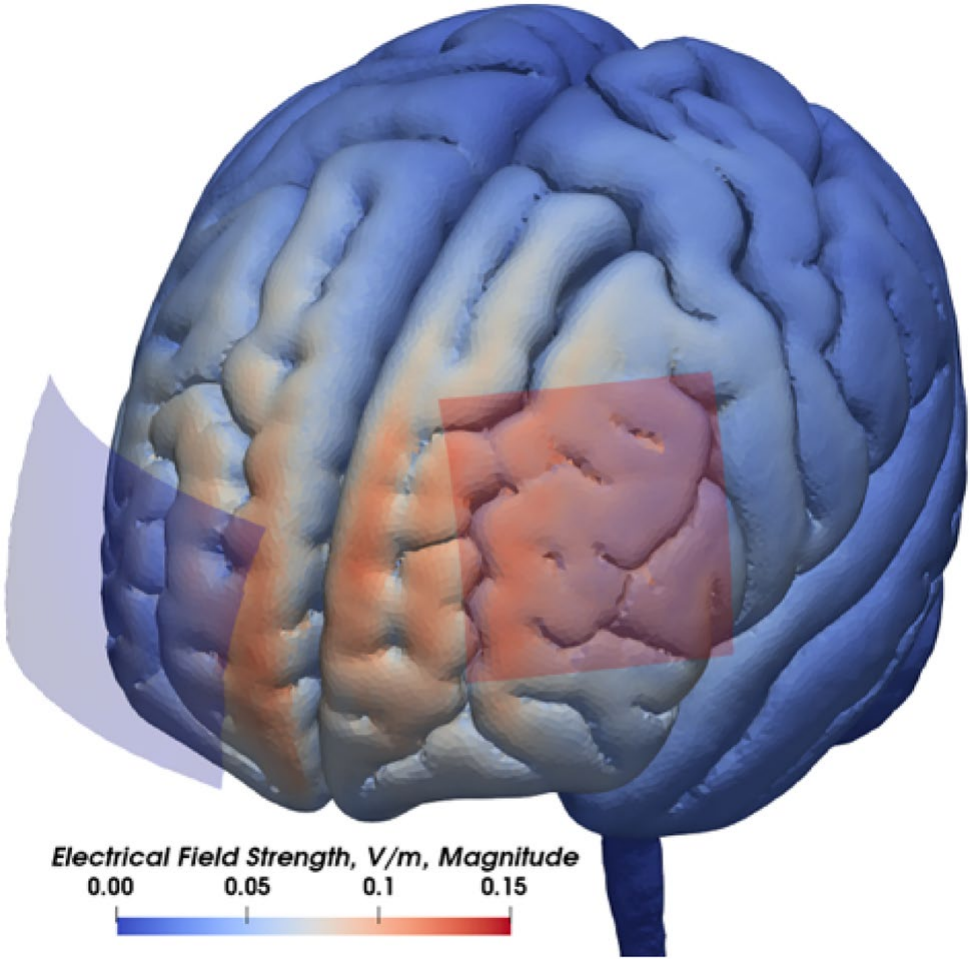
Modeling of field strength with SimNIBS 2.1 and visualization with ParaView (Kitware Inc.) under the active and reference electrode. We aimed at optimization of stimulating left dorsolateral prefrontal cortex

Bipolar tDCS was delivered by a battery-driven DC stimulator (neuroConn, Ilmenau, Germany) with 1 mA and administered using two MRI-compatible rubber electrodes which were attached to the subject’s head with Ten20® conductive Neurodiagnostic Electrode Paste (Weaver and Company, USA). One electrode was sized 5 × 5 cm resulting in a sufficient and tolerable current density at 0.04 mA/cm^2^. This electrode was placed above the target region F3 (according to the EEG international 10–20 system) corresponding to the left DLPFC and served, depending on the stimulation setting, as anode (anodal tDCS) or cathode (cathodal tDCS). The reference electrode was sized 5 × 7 cm, so that it was less efficient as compared to the active electrode [20]. We used the right supraorbital region as reference, at least 4 cm from the other electrode [21]. We primarily relied on the meta-analysis by Hill et al. and aimed for the most frequently applied parameters in tDCS studies in working memory (stimulation strength: 1 mA, stimulation strength: 20 min, anode location: F3) [5]. Using modeling with SimNIBS 2.1, this setup showed an optimal field strength around our target region in DLPFC (Fig. 2) [22].

 Functional magnetic resonance imaging data were acquired on a Siemens Magnetom Verio 3 Tesla scanner equipped with a standard 32-channel head coil. The setup used for stimulation and concomitant scanning can be seen in Fig. 1A and is further explained in the supplement. 

We used a classical task-based fMRI approach in order to find differences in local activation. Subjects were measured during the performance of an *n*-back task [14, 23]. During this task, subjects were seeing digits from zero to 9 for 500 ms with an inter-stimulus interval of 300 ms while lying supine inside the scanner. A task-block consisted of 22 stimuli with three target stimuli (Fig. 1B). Overall, there were 12 blocks (six 0-back and six 2-back blocks) resulting in a task duration of 10 min. We recorded reaction times and accuracy according to Snodgrass & Corwin (dprime = *z* (hit)—*z* (false positive)) [24]. During the resting-state scan, participants were instructed to try to think of nothing in particular and to keep their eyes open and focused on a white fixation cross against a black background. Scanning parameters can be found in Supplement. For anatomical reference, a high-resolution MPRAGE was used.

### Functional MRI analysis

Preprocessing of MRI data was realized using FMRIP Software Library FSL [25] and Advanced Normalization Tools ANTs [26] for spatial transformations. As described before [27, 28], these applications were combined in an established pipeline (https://github.com/NeuroanatomyAndConnectivity/pipelines/tree/master/src/lsd_lemon) implemented in Nipype [29]. Preprocessing of structural T1-weighted images was realized by masking out the background of each subject’s high-resolution anatomical image. Functional preprocessing steps included spatial transformations comprising realignment, distortion correction and co-registration to the structural high-resolution image, furthermore denoising, band-pass filtering (0.01–0.1 Hz), spatial smoothing with a 6-mm full width at half maximum (FWHM) kernel and normalization to standard space (MNI 152).

### Seed-based connectivity analysis

Seed-based connectivity tested the correlation between the left DLPFC time series, where stimulation took place, with the time series of all the other voxels in the brain. The left DLPFC seed region was defined by a sphere of 8 mm at the coordinate − 34, 26, 44 in MNI space. This coordinate was derived from Keeser et al. who converted the F3 position in the EEG international 10–20 system, defined as their region of interest in the context of anodal tDCS stimulation of the left DLPFC, into MNI coordinates [30]. For the seed-based connectivity analysis, we used the in-build ICA-independent component analysis of SPM12 to extract BOLD signal from our seed region that is then introduced in the design matrix on a single subject level. Individual seed-based connectivity was assessed by setting up individual GLMs including the dlPFC time series and motion regressors as well nuisance regressors including signals from cerebrospinal fluid (CSF) and white matter. On the second level, we conducted a flexible factorial ANOVA analysis and compared the anodal with the cathodal as well as sham stimulation condition using appropriate F-contrast on the whole-brain level. For a volume of interest (VOI) approach, we extracted mean beta values representing the connectivity from the seed in the left DLPFC to the four VOIs. We extracted the mean from VOIs in the fronto-parietal network including left DLPFC, right DLPFC, left parietal cortex (PC) and right PC [31].

### Dynamic causal modeling of resting-state fMRI

To provide information on directionality within the fronto-parietal network and for comparing effective connectivity between the different stimulation conditions (anodal and cathodal vs. sham), we applied dynamic causal modeling (DCM) [32]. As it is especially crucial to identify meaningful signals from background noise in resting-state fMRI, we used DCM as a further hypothesis-driven approach. In contrast to a generative approach of testing several different models, we decided to use a neurobiological plausible model (a full model of the fronto-parietal network) to further investigate how dynamics in different neuronal populations are influenced during manipulation, in this case the noninvasive stimulation.

Our DCM model included bilateral dlPFC and inferior parietal cortex (IPC) and comprised bilateral intrahemispheric and interhemispheric connections, which is shown schematically in Supplementary Figure F1. Both autoconnectivity within the regions and interregional connectivity parameters were assessed. Time series were extracted from separate general linear model (GLM) in order to remove variance related to movement parameter, and CSF and white matter regressors were used to clean the data from noise. These individual GLMs were then used to extract the time series of 4 different VOIs, which comprised the DLPFC bilaterally and the inferior parietal cortex (IPC) bilaterally [31]. The coordinates for the 4 VOIs were based on a meta-analysis on functional activation during the n-back task by Owen et al. 2005 [12] and converted from Talairach to MNI space using the MNI2tal tool embedded in BioImage Suite [33]. VOIs were defined as spheres with a radius of 8 mm around the coordinates (− 46 19 22) for left DLPFC, (41 31 30) for right DLPFC, (− 36 –53 43) for left IPC and (39 –51 40) for right IPC. We assessed the evidence for our model parameters using a one-state, bilinear, deterministic DCM without modulatory input. Due to the within-subject design, there was no need to apply spectral DCM, as this was intended primarily to avoid problems due to potential differences in neuronal activity between groups in a study [34].

We used R [35] and afex [36] to perform a linear mixed effects analysis of the relationship between connectivity and condition. As fixed effects, we entered stimulation condition and connection, as random effects we modeled the intercepts for each subject. Visual inspection of residual plots did not reveal any obvious deviations from homoscedasticity or normality. P values were obtained by repeated-measures ANOVA of the full model. To assess connectivity changes in specific connections, we performed post hoc pairwise comparisons using lsmeans.

### Task-based activation

Task-based activation was evaluated using the standard two-level approach [37] as implemented in SPM12. The first-level GLM modeled the 2-back and 0-back condition as block conditions as well as the instruction cue before each block for all three stimulation sessions. In addition, the six realignment parameters were included as regressors. On the second level, we calculated a repeated-measures ANOVA with the within-subject factors task (2-back, 0-back) and stimulation (anodal, cathodal, sham).

## Results

All participants tolerated the stimulation well. Reported side effects during stimulation included slight tingling in about half of the measurements (see Supplementary Table S1 for further details on blinding efficacy and side effects depending on stimulation condition).

### Resting-state fMRI

Whole brain results from seed in region of stimulation. We found a main effect of stimulation on a whole-brain level in the cerebellum (F(2.34) = 12.76, *p*(uncorr) = 10 × 10^–14^ at 14 –56 –36) which did not survive whole-brain FWE correction p(FWE-corr) = 0.714.

Seed-based analysis with extracted values from VOIs. The mean of the connectivity across regions was estimated at 0.19 (SD = 0.15). A one sample t test revealed that the connectivity was significantly different from zero (*t* = 24.68 (df = 419), p < 0.001).

**Fig. 3:**
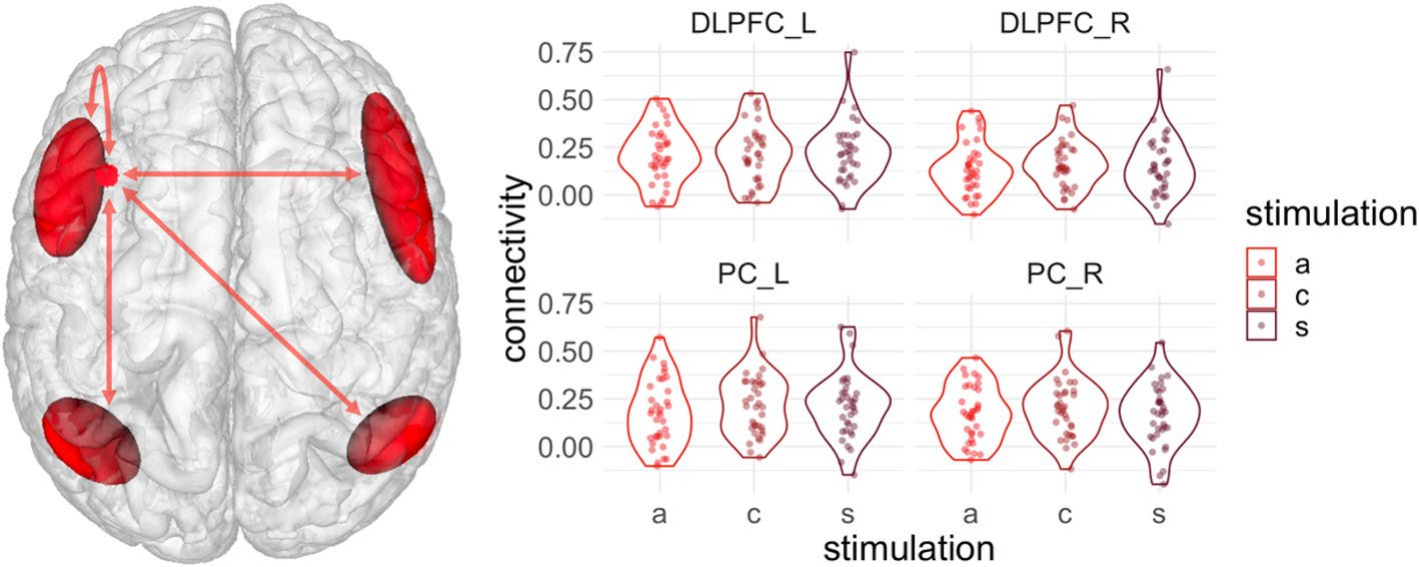
Modeling of seed-based connectivity. The left panel represents an illustration of the left dlPFC seed region (red dot) and the VOIs used to define the target region within the fronto-parietal network. From the left DLPFC, the point of stimulation, we calculated correlations to itself and to each region. In the right panel, we show connectivity parameters from seed-based analysis for each stimulation condition (*a*  anodal, *c* cathodal and *s* sham)

We conducted a repeated-measures ANOVA with the within-subject factors region of interest and stimulation condition. There was no significant main effect of stimulation (F(1.84, 62.59) = 1.19, *p* = 0.31) (Fig. 3). However, we did find a main effect for the investigated region (F(2.22, 75.39) = 5.03, *p* = 0.01) as well as an interaction between region of interest and stimulation (F (4.33, 147.38) = 2.89, *p* = 0.02).

Concerning the interaction effect, post hoc tests reveal that in left PC the anodal stimulation decreases connectivity with the left DLPFC (estimate = − 0.04, *t* = − 2.22, *p* = 0.03) as compared to cathodal stimulation and we observed a trendwise decrease vs. sham stimulation (estimate = − 0.03, *t *= − 1.72, *p* = 0.09). In right PC, we estimate a trend for a higher coupling with the left DLPFC during cathodal stimulation (estimate = − 0.04, *t* = − 1.93, *p* = 0.057) as compared to sham (see Supplementary Table S2). Further post hoc tests revealed overall different correlations depending on the region investigated, mainly due to left DLPFC revealing the strongest association putatively due to proximity to our seed region.

DCM. Autoconnectivity. The estimation of the autoconnectivity parameters of the 4 different VOIs showed the expected negative values (according to the underlying negative feedback function) that were different from zero (mean = − 0.06, SD = 0.01, *t* (419) = − 114, *p* < 2*10^–16^). (Supplementary Table S4).

In a second step, we tested whether the parameters were influenced by the stimulation condition. A repeated-measures ANOVA was conducted, which showed no significant change in connectivity (main effect of stimulation: F(1.98, 67.41) = 0.01, *p* = 0.99), nor region by stimulation interaction (F(4.65, 157.96) = 0.71, *p* = 0.6).

Concerning the eight interregional connectivities (fronto-parietal network interhemispherically, Supplementary Figure F1A), the connectivity parameters were all in the positive range (mean = 0.06, SD = 0.01, *t*(839) = 125 *p* < 2 × 10^–16^), for descriptives see Supplementary Table S5.

The repeated-measures ANOVA for the effect of stimulation showed no main effect on interregional connectivity (F (1.87, 63.68) = 0.94, *p* = 0.39). We found no interaction between connection and stimulation (F (4.51, 153.19) = 0.65, *p* = 0.65).

### Task-based fMRI

Whole-brain analysis revealed significant BOLD effects during WM performance in the known fronto-parietal network with a maximum effect in the right dorsolateral prefrontal cortex (*T* = 12.627, *p* = 2.672 × 10^–9^; *x* = 42; *y* = 34; *z* = 30, Supplementary Figure F2A and B and Supplementary Table S1). On the whole-brain level and on extracted parameters from our regions of interest, we tested whether there was a significant effect of stimulation on WM-related activation with a repeated-measures ANOVA. We found no significant effect of stimulation on the whole-brain level at *p* < 0.001 as well as on extracted parameters from our regions of interest (*F* = 0.22; df = 2, 70; *ε*^2^ = 0.001; *p* = 0.802, Supplementary Figure F2C). 

### Behavioral results

Reaction times were significantly different between the task conditions (0-back vs. 2-back, *F* = 320.206; df = 1, *p *< 0.001). We found no significant effect of stimulation on reaction times in the n-back task (F = 0.393, df = 2, p = 0.677). Accuracy as measured with dprime showed a significant task effect comparing 0-back and 2-back (*F* = 342.014, df = 1, *p* < 0.001); however, we found no significant effect of stimulation (*F* = 1.773, df = 2, *p* = 0.177).

A short summary of the results can be found in Table 1. A more thorough listing can be found in Supplementary Table S7.

**Table 1:** Short results summary of analysis methods and effects of stimulation after tDCS over left DLPFC

Analysis method	Effect
Whole-brain results	Main effect of stimulation on a whole-brain level in the cerebellum at xyz = 14-56-36 (not significant after whole-brain FWE correction)
Seed-based analysis in a ROI-to-ROI analysis in the fronto-parietal network	Interaction effect between region of interest and stimulation: anodal vs. cathodal tDCS: decreased connectivity between left DLPFC and left PC cathodal vs. sham tDCS: Trend for higher coupling between left DLPFC and right PC
Autoconnectivity	No significant effect
Interconnectivity (fronto-parietal network interhemispherically)	No significant effect
Whole-brain analysis	No significant effect of stimulation
Regions of interest	No significant effect of stimulation
Behavioral results	No significant effect of stimulation

## Discussion

Although noninvasive brain stimulation plays a viable role in detecting and modulating neural processes underlying higher cognitive functions, there is limited evidence on how tDCS might induce neuronal effects as measured by functional and effective connectivity changes associated with working memory networks.

Our goal was to answer the question whether and how anodal and cathodal stimulation in comparison with sham influences the fronto-parietal network in healthy subjects. Our main hypothesis was that anodal tDCS would increase fronto-parietal connectivity, as a neural basis for previously described tDCS effects on task performance.

We indeed found a significant stimulation by region interaction in the seed-based ROI-to-ROI resting-state connectivity; however, we could not show any effect on effective connectivity during rest. We did find robust fronto-parietal activation during task performance; however, stimulation showed no effect on the BOLD signal nor on working memory performance. This negative finding in stimulation effect on task activation might be due to limitations in the studied population and in study design that we will discuss in the following paragraphs:

Given the very homogenous high performing young sample, we might have missed significant behavioral effects due to potential ceiling effects, leading to limited possibilities to capture underlying neurobiological markers. Another possible explanation for the missing effect on task-based activation and performance may be that we measured task-based activation after stimulation, so that we might have missed direct stimulation effects. Online effects measured during rest were recorded during stimulation; however, stimulation intensity was apparently too low, or anode size was too big to reveal lasting after-effects on task-based activation and task performance after stimulation. Higher intensities and more focused currents might also have a larger and more lasting impact [38, 39]; however, novel studies show also nonlinear effects given putative effects of compensatory mechanisms at higher intensities [40].

Another limitation of the study is that we did not test for other electrode placements. It has been shown that the choice of the reference region influences tDCS current flow and effects [2, 21]. Additionally, through possible simultaneous modulation of related, but unexamined cognitive functions other than working memory after stimulation over the DLPFC, heterogeneous results could be produced [41]. Further research is warranted to evaluate different options in the setup of electrode placement, including the significance of an active control in addition to a sham control group [42].

Our seed-based connectivity analysis on a whole-brain level revealed an activation in the cerebellum, although this did not survive multiple comparisons. This could be a hint for possible long-range effects in a previously identified specific fronto-cerebellar circuit associated with the default network and with nonmotor functions [43–45].

The analysis of our VOI-based approach suggested that in the left parietal cortex the anodal stimulation tends to decrease connectivity as compared to cathodal stimulation. We also observed a trendwise decrease vs. sham stimulation. This seems surprising as previous studies have shown an increase in functional connectivity after anodal tDCS, possibly through synchronization of neuronal populations as main mechanism [46]. Polania et al. [47] recorded EEG measurements before and after anodal tDCS over the primary motor cortex, combined with cathodal tDCS of the contralateral frontopolar cortex, in resting state and during voluntary hand movements. They found that stimulation during resting state induced a significant synchronization increase within frontal electrodes in the gamma, alpha and beta bands. Presumably, modification of membrane potentials induced by tDCS is also determined by cortical layer and spatial orientation of stimulated neurons [48]. Polania et al. discussed whether the cathodal frontopolar-placed electrode might have induced a constant negative electrical field during the stimulation which might have provoked a hyperpolarization of the neurons and consequently increased the synchronization of the spontaneous activity over the cathodal stimulated region.

Another previous finding to consider is that resting-state functional coupling was decreased during anodal tDCS compared with sham tDCS, which was interpreted as increased efficiency given changes in GABA levels [49]. The authors interpret the increased coupling that occurs during aging might be inverted through the anodal tDCS. In line with these findings in our sample, we did observe a decreased coupling under anodal tDCS; however, given that we did not show evidence for increased efficiency (i.e., increased performance) this finding needs further investigation.

In summary, in the literature there is a significant amount of heterogeneity primarily due to differences in setups (e.g., stimulation parameters, state dependency, i.e., online vs. offline stimulation). Our findings add to the increasing evidence for tDCS-induced connectivity changes, although in our study the tDCS-induced connectivity changes are most probably rather unspecific with no clear behavioral equivalents.

DCM offers the generative modeling of directional connectivity in a specific network which we implemented. While resting-state fMRI remains an important and elucidating noninvasive method to deconstruct brain networks, it might not be able to examine tDCS-modulated cognitive performance. As at least one previous study indicated, the activation of a network through appropriate tasks might make it more sensitive to tDCS modulation [50]. Our DCM analysis indicated that our analysis yielded meaningful estimates (negative values for autoconnectivity parameters and positive values for interregional connectivity, all different from zero). However, the comparison of parameter estimates showed no significant effect of stimulation.

Previous research [51] which showed an increase in resting-state connectivity of the left inferior frontal gyrus (IFG) during anodal tDCS used graph-based data analysis approach (eigenvector centrality mapping, ECM). This method uses spectral coherence patterns across the brain to define connectivity of central cortical nodes [52]. The IFG showed significantly higher eigenvector centrality values as compared to sham stimulation, and additional major hubs overlapping with the language network showed significant differences depending on stimulation.

It has been shown repeatedly that prefrontal tDCS could increase executive function such as working memory in patients with various neurological and psychiatric disorders [53–57]. In healthy cohorts, however, these effects are not consistent [58–60]. Mancuso et al. [59] showed in a meta-analysis that anodal tDCS of the left DLPFC in combination with WM training could significantly increase WM performance. After correction for publication bias, there was no effect of stimulation alone. Given the various cognitive functions potentially modulated by tDCS over DLPFC (e.g., working memory, visuospatial memory, inhibition, emotion regulation among others), a possible simultaneous modulation of those functions might lead to sometimes contradictory results [41]. Additionally, as a recent study using computational modeling of tDCS-induced electric fields could show, there seems to exist a large inter-individual variation in electric field intensity distribution and possibly related effects on glutamate levels varying with electric field strength [61]. Beyond heterogeneous study designs, cohorts and stimulation protocols, small study size might be a limitation. Although we took care in powering our study, still the limited number of participants could explain the null findings with a power of 80%.

In conclusion, we found that anodal tDCS over the DLPFC could decrease functional connectivity with the parietal cortex, indicating altered synchronization. However, we could not find any indication for altered task activation and effective and directed connectivity due to tDCS stimulation. This might substantiate the theoretical framework which sees tDCS as a tool to foster neurological learning mechanisms but not static performance retrieval, thus benefiting neuropsychiatric populations and learning processes.

### Supplementary Information

Below is the link to the electronic supplementary material.

Supplementary file1 (DOCX 1771 KB)

## Data Availability

Raw data were generated at Max-Planck Institute for Human Cognitive and Brain Sciences, Leipzig, Germany. Derived data supporting the findings of this study are available from the corresponding author JK on request.
