# Supplementary material for: Influence of noninvasive brain stimulation on connectivity and local activation: a combined tDCS and fMRI study
Source: Eur Arch Psychiatry Clin Neurosci. 2023 Aug 19;274(4):827–35. doi: 10.1007/s00406-023-01666-y (PMC11127864; doi:10.1007/s00406-023-01666-y)
Supplement: Supplementary file 1 — Supplementary file1 (DOCX 1771 KB) [file 406_2023_1666_MOESM1_ESM.docx]

Supplementary Material

**Journal:** European Archives of Psychiatry and Clinical Neuroscience

**Influence of non-invasive brain stimulation on connectivity and local activation: a combined tDCS and fMRI study**

Luise Victoria Claaß* Annika Hedrich*, Janis Reinelt, Bernhard Sehm, Arno Villringer, Florian Schlagenhauf, Jakob Kaminski

*these authors contributed equally to this work

**Corresponding author:**

Jakob Kaminski, M.D.

Department of Psychiatry and Psychotherapy

Charité–Universitätsmedizin Berlin, Campus Mitte

Charitéplatz 1, 10117 Berlin, Germany

Tel. +49-30-450 517095

E-mail: [jakob.kaminski@charite.de](mailto:jakob.kaminski@charite.de)

Intro:

We here present additional information on methods and provide supplementary results from our analysis.

Methods:

The rubber electrodes were equipped with MR-compatible 5,6-kΩ resistors in each wire to avoid sudden temperature increases due to induction voltages from radio frequency pulses in the MRI-scanner, as described previously [1]. The stimulator was located in the control room and set at 1mA. The cables connecting electrodes and stimulator passed on their way through the MR cabin wall a radio frequency filter to reduce possible artifacts. Two filter boxes (Neuroconn, Ilmenau) were positioned between electrodes and stimulator, one of them inside the MR cabin, the other one immediately outside.

Before the actual scan, a measurement of the impedance was performed. If the impedance was above 10 kΩ, the electrodes were re-attached, until the impedance was below the limit.

Modeling of field strength was conducted using SimNIBS 2.1 [2] and visualized by ParaView [3]. As head model served the MNIHead standard model (which is based on the FSL MNI152 model) and which is supplied within SimNIBS.

High resolution structural MR images were acquired using an MP2RAGE sequence: sagittal acquisition orientation, one 3D volume with 176 slices, repetition time (TR) = 5000 ms, TE = 2.92 ms, TI1 = 700 ms, TI2 = 2500 ms, FA1 = 4°, FA2. = 5°, pre-scan normalization, echo spacing = 6.9 ms, bandwidth = 240 Hz/pixel, FOV = 256 mm, voxel size = 1 mm isotropic, GRAPPA acceleration factor 3, slice order = interleaved, duration = 8 min 22 s [4].

Continuous resting-state BOLD-fMRI was acquired with the following parameters: resolution, 2,3x2,3x2,3 mm^3^; TR=1400ms; TE=30ms; flip angle 69°; interleaved acquisition, field-of-view (FoV) 202x202 mm^2^; acquisition matrix 64x64, total number of volumes 850. Functional images were acquired in axial orientation. For each individual resting-state scan field maps were gathered (TR=493ms, TE1=5.19ms, TE2=7.65 ms, flip angle=60°, voxel size=4.0 mm isotropic, 30 slices) to correct for magnetic field inhomogeneities.

For measuring functional activation during task, time to echo was set at three milliseconds with a repetition time at 2000 milliseconds, flip angle was set at 90°.

Results:

Resting-state fMRI:

Seed-based analysis:

In Table S3 we show pair-wise comparisons for each region. Given the least squares means approach that assumes homogeneous error structures, and since the design is evidently balanced, the standard errors are the same.

DCM analysis:

As mentioned in the main results we did not find a main effect for stimulation or an interaction between stimulation and connection. However, we did find a main effect for the investigated connection (F (2.92,99.27) =3.26, p=0.03). This is primarily due to the fact that several post-hoc tests comparing connections result in significant differences between the respective estimates (see Table S6). We did not have a specific hypothesis for differences in the connections therefore we do not further interpret this effect.

Task-based fMRI:

In Table S2 we present significant differences in activation contrasting 2-back vs. 0-back.

We screened all single-subject maps for significant voxels within our VOI and found significant p<0.05 uncorrected voxels (on a single subject level) in all subjects. Additionally, as a sanity check, we extracted parameters from a larger sphere with a 16mm diameter. As expected, we found significantly correlated parameters between the 8mm and the 16mm sphere ranging from R=0.46 from DLPFC_L and R=0.62 in PC_R. We then calculated the repeated measures ANOVA for the data from the wider sphere and found similar region effects but no effect of stimulation F = 0.98; df = 2, 70; ε^2^ = 0.01; p = 0.379.


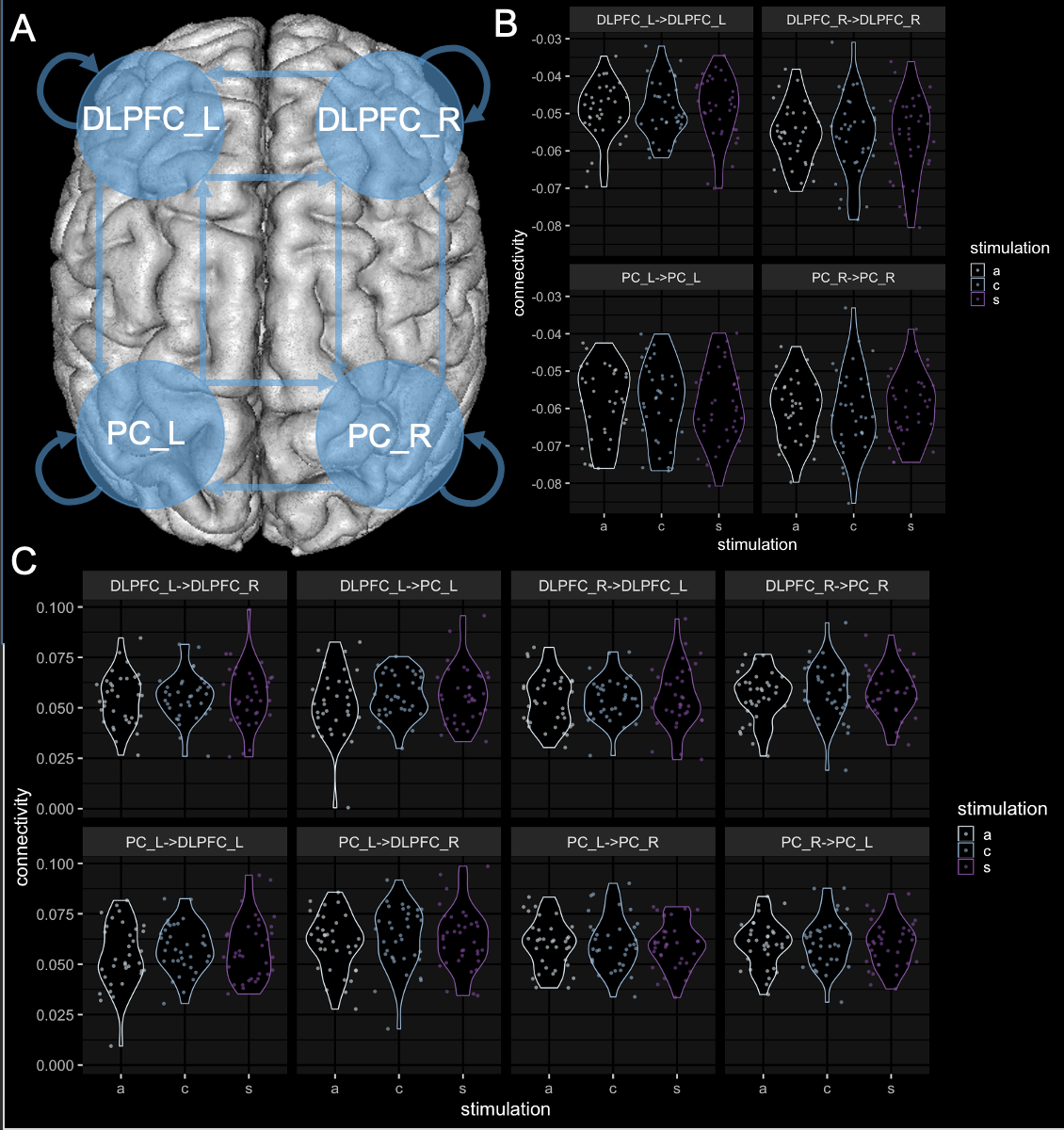


Figure F1. **A)** Illustration of DCM analysis to evaluate directed effective connectivity **B)** Estimation of interregional connectivity parameters. Containing all of the eight available interregional connections, a mixed-models ANOVA was calculated to determine whether there was a difference between stimulation conditions. We could show a significant effect for connection, but no interaction effect between connection and stimulation condition, nor an overall stimulation effect on interregional connectivity. Stimulation condition (a=anodal, c=cathodal and s=sham)


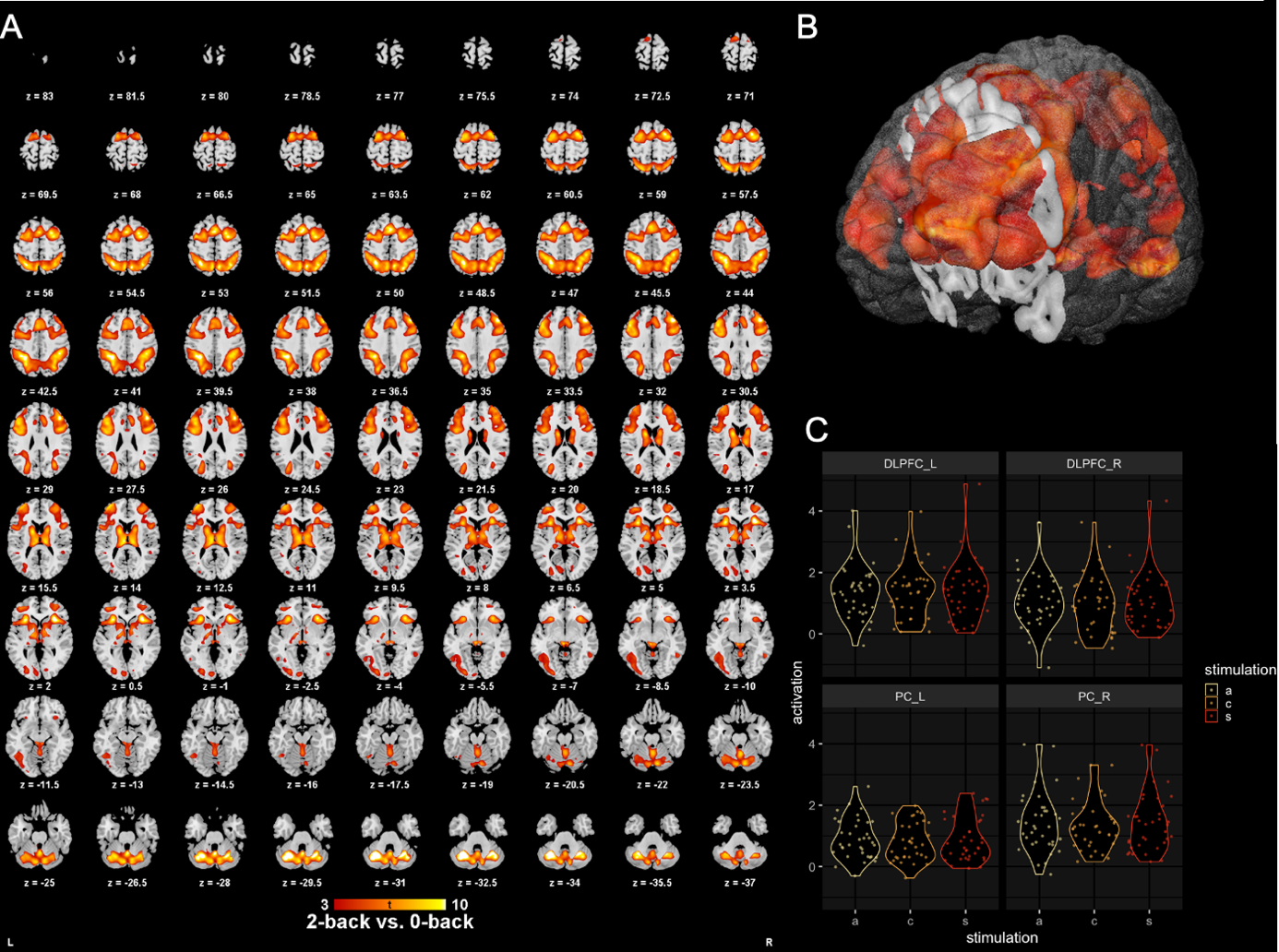


Figure F2. **A)** We here present axial slices with activation maps contrasting 2-back vs. 0-back **B)** 3-D rendering of local activation of fMRI measurements during 2-back vs. 0-back task. Fronto-parietal networks are bihemispherically activated **C)** There was no significant difference in activation patterns between stimulation conditions (a=anodal, c=cathodal and s=sham), compared in each region separately.

Blinding efficiency:

In a questionnaire that was administered after the final measurement, we asked the subjects whether they could differentiate between active and sham stimulation. This was possible for 86% and 81% of participants during anodal and cathodal stimulation, respectively, but only for 47% during sham stimulation. The responses of the related questionnaire items are shown in Table S1. We also recorded the subjects' certainty on having answered correctly on a scale from 0 to 10 in our questionnaire. Response reliability and stimulation type did not significantly interact in a repeated-measures ANOVA (F(2,70) = 0.45; p = 0.637).

Table S1. Blinding and side effects depending on stimulation condition.

|  | **Percentage (%)** |
| --- | --- |
| **Subjectively received active stimulation** | **73** |
| anodal | 86 |
| cathodal | 81 |
| sham | 53 |
| **Stimulation condition correctly detected** | **71** |
| anodal | 86 |
| cathodal | 81 |
| sham | 47 |
| **Perceived any side effects** | **53** |
| anodal | 56 |
| cathodal | 58 |
| sham | 44 |
| **Certainty of having answered correctly** | **Mean (SD)** |
| anodal | 7,47 (±2,09) |
| cathodal | 7,31 (±2,78) |
| sham | 7,11 (±2,55) |
|  | |

Estimated active stimulation and stimulation condition interacted significantly with each other (F(2,70) = 5.59; p = 006). A post-hoc analysis showed that subjects were significantly more likely to suspect active stimulation in the anodal and cathodal sessions compared with sham (anodal vs. sham (estimate = -0.33; t = -3.12; p = 0.003), cathodal vs. sham (estimate = 0.28; t = 2.60; p = 0.011)).

This might indicate that blinding of the participants was not efficient enough. Because in active tDCS sensory side effects such as tingling and itching are more frequent and more severe than in sham tDCS, it has been discussed that current methods of sham stimulation may not be adequate enough to serve as control condition [5]. Since sham procedures are essential due to the placebo response observed in non-invasive brain stimulation trials [6] a potential blinding bias in our study could have led to inconclusive results. On the other hand, meta-analytically there has been little evidence of blinding bias in trials with objective outcomes but substantial bias in trials with subjective outcome measures [7]. Overall, this underlines the urgent need for more effective and comparable sham tDCS protocols for future trials.

Table S2. Task-based fMRI activation peaks for 2-back vs. 0-back contrast across all participants.

| **p(FWE-corr)** | **T** | **equivZ** | **x** | **y** | **z** | **region** |
| --- | --- | --- | --- | --- | --- | --- |
| 6.40E-07 | 10.27 | 6.83 | -36 | 58 | 12 | Middle Frontal Gyrus |
| 1.80E-03 | 7.14 | 5.51 | 32 | 52 | 20 | Superior Frontal Gyrus |
| 1.85E-03 | 7.13 | 5.51 | 40 | 48 | 22 | Middle Frontal Gyrus |
| 3.45E-03 | 6.88 | 5.38 | -40 | 48 | 2 | Inferior Frontal Gyrus |
| 2.67E-09 | 12.63 | 7.58 | 42 | 34 | 30 | Middle Frontal Gyrus |
| 6.69E-09 | 12.21 | 7.46 | 30 | 22 | 4 | Insula |
| 2.04E-06 | 9.80 | 6.66 | -2 | 22 | 46 | Cingulate Gyrus |
| 2.24E-07 | 10.70 | 6.98 | -30 | 20 | 4 | Insula |
| 4.23E-04 | 7.74 | 5.80 | 48 | 18 | 4 | Inferior Frontal Gyrus |
| 1.51E-07 | 10.86 | 7.04 | 2 | 16 | 50 | Medial Frontal Gyrus |
| 2.50E-05 | 8.84 | 6.29 | 44 | 10 | 30 | Inferior Frontal Gyrus |
| 3.57E-08 | 11.47 | 7.23 | -2 | 10 | 54 | Superior Frontal Gyrus |
| 2.63E-07 | 10.63 | 6.96 | 24 | 8 | 54 | Superior Frontal Gyrus |
| 4.68E-06 | 9.48 | 6.54 | 34 | 4 | 60 | Middle Frontal Gyrus |
| 7.17E-04 | 7.52 | 5.70 | 18 | 4 | 66 | Middle Frontal Gyrus |
| 2.56E-05 | 8.83 | 6.28 | 18 | 4 | 14 | Extra-Nuclear |
| 8.00E-07 | 10.18 | 6.80 | 14 | 0 | -2 | Lentiform Nucleus |
| 8.63E-07 | 10.15 | 6.79 | -16 | 0 | 16 | Extra-Nuclear |
| 2.18E-06 | 9.78 | 6.65 | -16 | -6 | 4 | Lentiform Nucleus |
| 2.54E-06 | 9.72 | 6.63 | -6 | -18 | 14 | Thalamus |
| 3.63E-06 | 9.58 | 6.58 | 12 | -22 | 14 | Thalamus |
| 3.30E-06 | 9.61 | 6.59 | -4 | -26 | -8 | undefined |
| 3.50E-07 | 10.51 | 6.92 | 42 | -38 | 42 | Inferior Parietal Lobule |
| 4.31E-08 | 11.39 | 7.21 | -32 | -44 | 38 | Sub-Gyral |
| 1.51E-07 | 10.86 | 7.04 | -40 | -44 | 40 | Inferior Parietal Lobule |
| 5.11E-09 | 12.33 | 7.49 | -30 | -54 | 50 | Superior Parietal Lobule |
| 8.68E-10 | 13.15 | 7.72 | 24 | -60 | -32 | Culmen |
| 1.21E-09 | 13.00 | 7.68 | -24 | -60 | -30 | Declive |
| 3.08E-08 | 11.54 | 7.25 | 10 | -62 | 52 | Precuneus |
| 1.92E-09 | 12.78 | 7.62 | -36 | -66 | -30 | Declive |
| 8.43E-08 | 11.11 | 7.12 | 28 | -66 | 48 | Superior Parietal Lobule |

Table S3. Seed based connectivity: post-hoc analysis with pair-wise comparisons (DLPFC_L = left dorsolateral prefrontal cortex, DLPFC_R = right dorsolateral prefrontal cortex, PC_L = left parietal cortex, PC_R = right parietal cortex)

| **contrast** | **region** | **estimate** | **SE** | **df** | **t ratio** | **p value** |
| --- | --- | --- | --- | --- | --- | --- |
| a - s | DLPFC_L | -0.0173 | 0.019 | 100 | -0.89 | 0.374 |
| a - c | DLPFC_L | -0.0111 | 0.019 | 100 | -0.57 | 0.569 |
| s - c | DLPFC_L | 0.0062 | 0.019 | 100 | 0.32 | 0.748 |
| a - s | DLPFC_R | -0.0097 | 0.019 | 100 | -0.50 | 0.617 |
| a - c | DLPFC_R | -0.0249 | 0.019 | 100 | -1.28 | 0.203 |
| s - c | DLPFC_R | -0.0151 | 0.019 | 100 | -0.78 | 0.438 |
| a - s | PC_L | -0.0334 | 0.019 | 100 | -1.72 | 0.088 |
| a - c | PC_L | -0.0431 | 0.019 | 100 | -2.22 | 0.029 |
| s - c | PC_L | -0.0096 | 0.019 | 100 | -0.50 | 0.621 |
| a - s | PC_R | 0.0082 | 0.019 | 100 | 0.42 | 0.673 |
| a - c | PC_R | -0.0292 | 0.019 | 100 | -1.50 | 0.136 |
| s - c | PC_R | -0.0374 | 0.019 | 100 | -1.93 | 0.057 |

Table S4. Autoconnectivity descriptives.

|  | **mean** | **sd** | **median** | **trimmed** | **mad** | **min** | **max** | **range** | **skew** | **kurtosis** | **se** |
| --- | --- | --- | --- | --- | --- | --- | --- | --- | --- | --- | --- |
| connectivity | -0.06 | 0.01 | -0.05 | -0.06 | 0.01 | -0.09 | -0.03 | 0.05 | -0.2 | -0.45 | 0.00 |

Table S5. Interregional connectivity descriptives.

|  | **mean** | **sd** | **median** | **trimmed** | **mad** | **min** | **max** | **range** | **skew** | **kurtosis** | **se** |
| --- | --- | --- | --- | --- | --- | --- | --- | --- | --- | --- | --- |
| connectivity | 0.06 | 0.01 | 0.06 | 0.06 | 0.01 | 0 | 0.1 | 0.1 | -0.03 | 0.43 | 0.00 |

Table S6: Post-hoc analysis with pair-wise comparisons of DCM interregional connection (rPFC = right dorsolateral prefrontal cortex, lPFC = left dorsolateral prefrontal cortex, rIPL = right inferior parietal lobule, lIPL = left inferior parietal lobule)

| **contrast** | **estimate** | **SE** | **df** | **t.ratio** | **p.value** |
| --- | --- | --- | --- | --- | --- |
| A_rPFC...lPFC - A_rPFC...rIPL | -0.00298 | 0.0022 | 238 | -1.356 | 0.17648 |
| A_rPFC...lPFC - A_lPFC...rPFC | -0.00090 | 0.0022 | 238 | -0.409 | 0.68302 |
| A_rPFC...lPFC - A_lPFC...lIPL | -0.00076 | 0.0022 | 238 | -0.345 | 0.73019 |
| A_rPFC...lPFC - A_rIPL...rPFC | -0.00767 | 0.0022 | 238 | -3.485 | 0.00059 |
| A_rPFC...lPFC - A_rIPL...lIPL | -0.00593 | 0.0022 | 238 | -2.694 | 0.00756 |
| A_rPFC...lPFC - A_lIPL...lPFC | -0.00225 | 0.0022 | 238 | -1.022 | 0.30779 |
| A_rPFC...lPFC - A_lIPL...rIPL | -0.00554 | 0.0022 | 238 | -2.515 | 0.01256 |
| A_rPFC...rIPL - A_lPFC...rPFC | 0.00208 | 0.0022 | 238 | 0.947 | 0.34468 |
| A_rPFC...rIPL - A_lPFC...lIPL | 0.00222 | 0.0022 | 238 | 1.010 | 0.31333 |
| A_rPFC...rIPL - A_rIPL...rPFC | -0.00469 | 0.0022 | 238 | -2.129 | 0.03428 |
| A_rPFC...rIPL - A_rIPL...lIPL | -0.00295 | 0.0022 | 238 | -1.338 | 0.18207 |
| A_rPFC...rIPL - A_lIPL...lPFC | 0.00073 | 0.0022 | 238 | 0.334 | 0.73895 |
| A_rPFC...rIPL - A_lIPL...rIPL | -0.00255 | 0.0022 | 238 | -1.159 | 0.24749 |
| A_lPFC...rPFC - A_lPFC...lIPL | 0.00014 | 0.0022 | 238 | 0.064 | 0.94937 |
| A_lPFC...rPFC - A_rIPL...rPFC | -0.00677 | 0.0022 | 238 | -3.076 | 0.00234 |
| A_lPFC...rPFC - A_rIPL...lIPL | -0.00503 | 0.0022 | 238 | -2.285 | 0.02318 |
| A_lPFC...rPFC - A_lIPL...lPFC | -0.00135 | 0.0022 | 238 | -0.613 | 0.54032 |
| A_lPFC...rPFC - A_lIPL...rIPL | -0.00464 | 0.0022 | 238 | -2.106 | 0.03624 |
| A_lPFC...lIPL - A_rIPL...rPFC | -0.00691 | 0.0022 | 238 | -3.139 | 0.00191 |
| A_lPFC...lIPL - A_rIPL...lIPL | -0.00517 | 0.0022 | 238 | -2.349 | 0.01966 |
| A_lPFC...lIPL - A_lIPL...lPFC | -0.00149 | 0.0022 | 238 | -0.677 | 0.49921 |
| A_lPFC...lIPL - A_lIPL...rIPL | -0.00478 | 0.0022 | 238 | -2.170 | 0.03102 |
| A_rIPL...rPFC - A_rIPL...lIPL | 0.00174 | 0.0022 | 238 | 0.791 | 0.42988 |
| A_rIPL...rPFC - A_lIPL...lPFC | 0.00542 | 0.0022 | 238 | 2.463 | 0.01450 |
| A_rIPL...rPFC - A_lIPL...rIPL | 0.00213 | 0.0022 | 238 | 0.970 | 0.33316 |
| A_rIPL...lIPL - A_lIPL...lPFC | 0.00368 | 0.0022 | 238 | 1.672 | 0.09585 |
| A_rIPL...lIPL - A_lIPL...rIPL | 0.00039 | 0.0022 | 238 | 0.179 | 0.85809 |
| A_lIPL...lPFC - A_lIPL...rIPL | -0.00329 | 0.0022 | 238 | -1.493 | 0.13677 |

Table S7: Overview of the effects of stimulation

| Method | Effect | Significance |
| --- | --- | --- |
| Resting-state fMRI | | |
| Whole brain results | Main effect of stimulation on a whole brain level in the cerebellum at xy = 14 -56 -36 | not significant after whole-brain FWE correction |
| Seed-based analysis in a ROI-to-ROI analysis in the frontoparietal network | Connectivity mean across regions = 0.19 (SD=0.15) | Connectivity significantly different from 0 (p<.001) |
|  | No significant main effect of stimulation |  |
|  | Main effect for the investigated region |  |
|  | Interaction effect between region of interest and stimulation | anodal vs. cathodal tDCS: decreased connectivity between left DLPFC and left PC  cathodal vs. sham tDCS:  Trend for higher coupling between left DLPFC and right PC |
| DCM | | |
| Autoconnectivity | All negative values | Probably due to underlying negative feedback function |
|  | No significant change in connectivity,  No region by stimulation interaction effect |  |
| Interconnectivity (fronto-parietal network interhemispherically) | All positive values (mean 0.06, SD=0.01) |  |
|  | No main effect of stimulation on interregional connectivity,  No interaction between connection and stimulation |  |
| Task-based fMRI | | |
| Whole-brain analysis | Significant BOLD effects during WM performance in the known fronto-parietal network, maximum effect in right DLPFC |  |
|  | No significant effect of stimulation at p<0.005 |  |
| Regions of interest | No significant effect of stimulation at p<0.005 |  |
| Behavioral results | Reaction times significantly different between task conditions |  |
|  | No significant effect of stimulation on reaction times in n-back task |  |
|  | 0-back vs. 2-back:  Significant task effect in Accuracy (measured with dprime) without effect of stimulation |  |

Bibliography

1. Antal, A., et al., *Transcranial direct current stimulation over the primary motor cortex during fMRI.* Neuroimage, 2011. **55**(2): p. 590-6.

2. Thielscher, A., A. Antunes, and G.B. Saturnino. *Field modeling for transcranial magnetic stimulation: A useful tool to understand the physiological effects of TMS?* in *2015 37th Annual International Conference of the IEEE Engineering in Medicine and Biology Society (EMBC)*. 2015.

3. Squillacote, A.H., *The Paraview Guide : A Parallel Visualization Application*. 2007: Clifton Park N.Y: Kitware.

4. Marques, J.P., et al., *MP2RAGE, a self bias-field corrected sequence for improved segmentation and T1-mapping at high field.* NeuroImage, 2010. **49**(2): p. 1271-1281.

5. Kessler, S.K., et al., *Differences in the experience of active and sham transcranial direct current stimulation.* Brain Stimulation: Basic, Translational, and Clinical Research in Neuromodulation, 2012. **5**(2): p. 155-162.

6. Razza, L.B., et al., *A systematic review and meta-analysis on placebo response to repetitive transcranial magnetic stimulation for depression trials.* Progress in Neuro-Psychopharmacology and Biological Psychiatry, 2018. **81**: p. 105-113.

7. Bikson, M., et al., *Safety of Transcranial Direct Current Stimulation: Evidence Based Update 2016.* Brain Stimul, 2016. **9**(5): p. 641-61.
